# Exercise during pregnancy protects adult mouse offspring from diet-induced obesity

**DOI:** 10.1186/s12986-015-0052-z

**Published:** 2015-12-18

**Authors:** Frederick Wasinski, Reury Frank Pereira Bacurau, Gabriel Rufino Estrela, Friederike Klempin, Aline Midori Arakaki, Rogerio Oliveira Batista, Fernando Francisco Pazello Mafra, Lucas Francisco Ribeiro do Nascimento, Meire Ioshie Hiyane, Lício Augusto Velloso, Niels Olsen Saraiva Câmara, Ronaldo Carvalho Araujo

**Affiliations:** Department of Biophysics, Federal University of São Paulo, Rua Pedro de Toledo, 669 9 andar, 04039-032 São Paulo, SP Brazil; Department of Medicine, Division of Nephrology, Federal University of São Paulo, 04023-900 São Paulo, SP Brazil; School of Arts, Sciences and Humanities, University of São Paulo, 03828-000 São Paulo, SP Brazil; Max Delbruck Center for Molecular Medicine, 13125 Berlin, Germany; Department of Internal Medicine, Faculty of Medical Sciences, State University of Campinas, Campinas, SP Brazil; Department of Immunology, Laboratory of Transplantation Immunobiology, Institute of Biomedical Sciences, University of São Paulo, 05508-900 São Paulo, SP Brazil

**Keywords:** Exercise during pregnancy, Physical exercise, Obesity in offspring

## Abstract

**Background:**

Physical exercise induces positive alterations in gene expression involved in the metabolism of obesity. Maternal exercise provokes adaptations soon after birth in the offspring. Here, we investigated whether adult mouse offspring of swim-trained mothers is protected against the development of the deleterious effects of high fat diet (HFD).

**Methods:**

Our study comprises two parts. First, female C57BL/6 mice were divided into one sedentary and one swim-trained group (before and during pregnancy, *n* = 18). In the second part, adult offspring (*n* = 12) of trained and sedentary mothers was challenged to HFD for 16 weeks. Notably, most of the analysis was done in male offspring.

**Results:**

Our results demonstrate that maternal exercise has several beneficial effects on the mouse offspring and protects them from the deleterious effects of HFD in the adult. Specifically, swimming during pregnancy leads to lower birth weight in offspring through 2 months of age. When subjected to HFD for 4 month in the adulthood, our study presents novel data on the male offspring’s metabolism of trained mothers. The offspring gained less weight, which was accompanied by less body fat, and they used more calories during daytime compared with offspring of sedentary mothers. Furthermore, we observed increased adiponectin expression in skeletal muscle, which was accompanied by decreased leptin levels and increased insulin sensitivity. Decreased interleukin-6 expression and increased peptide PYY levels were observed in sera of adult offspring of mothers that swam during pregnancy.

**Conclusions:**

Our results point to the conclusion that maternal exercise is beneficial to protect the offspring from developing obesity, which could be important for succeeding generations as well.

**Electronic supplementary material:**

The online version of this article (doi:10.1186/s12986-015-0052-z) contains supplementary material, which is available to authorized users.

## Background

Obesity is a risk factor for developing chronic diseases such as hypertension and diabetes in both industrial and developing countries. Overeating and eating disorders have become a worldwide epidemic. Obesity during pregnancy may represent a risk to the offspring by promoting higher birth weight and increasing the odds of developing obesity or type 2 diabetes in adulthood [1]. Conversely, low birth weight that results from insufficient food intake or smoking during pregnancy can lead to obesity, cardiovascular disease, renal dysfunction, or type 2 diabetes in offspring [2, 3].

Physical exercise is an important component of a healthy lifestyle. Lately, it has become attractive to prescribe mild physical exercise to pregnant women for a determined duration and intensity to minimize both fetal and maternal risks. The prescribed paradigm allows previously active women to maintain their exercise routine during pregnancy and reduces the rates of preeclampsia, gestational diabetes, heartburn and cesarean section in those who have never previously exercised, which may lead to better weight control [4]. Previous reports also indicate that physical activity during pregnancy can reduce the risk of delivering a large baby [5]. Yet, little is known about maternal exercise and the long-term consequences for offspring.

In animal models, offspring of trained mothers displayed positive organic adaptations soon after birth [6, 7]. The beneficial effect of maternal exercise is due to various physiological adaptations such as respiration and weight control in offspring. Exercise may also induce hormonal changes and alterations in gene expression [8]. More specifically, studies on maternal exercise in rodents, e.g., voluntary wheel running, revealed improved glucose homeostasis in adult offspring [9]. Yet, only few data exist on the metabolic consequences of exercise during pregnancy for mothers and their offspring, and such data varies between studies [10–13]. Swimming is one of the most moderate exercises for the entire body because it puts less weight on bones and ankles than other sports, and it can be continued during pregnancy. Thus, we hypothesize that sedentary offspring from mothers submitted to a regular exercise program could be protected from the deleterious effect of HFD during adulthood. Therefore, we investigated whether swimming during pregnancy could affect offspring and their predisposition to develop obesity in adulthood.

## Methods

### Animals

**Fig. 1:**
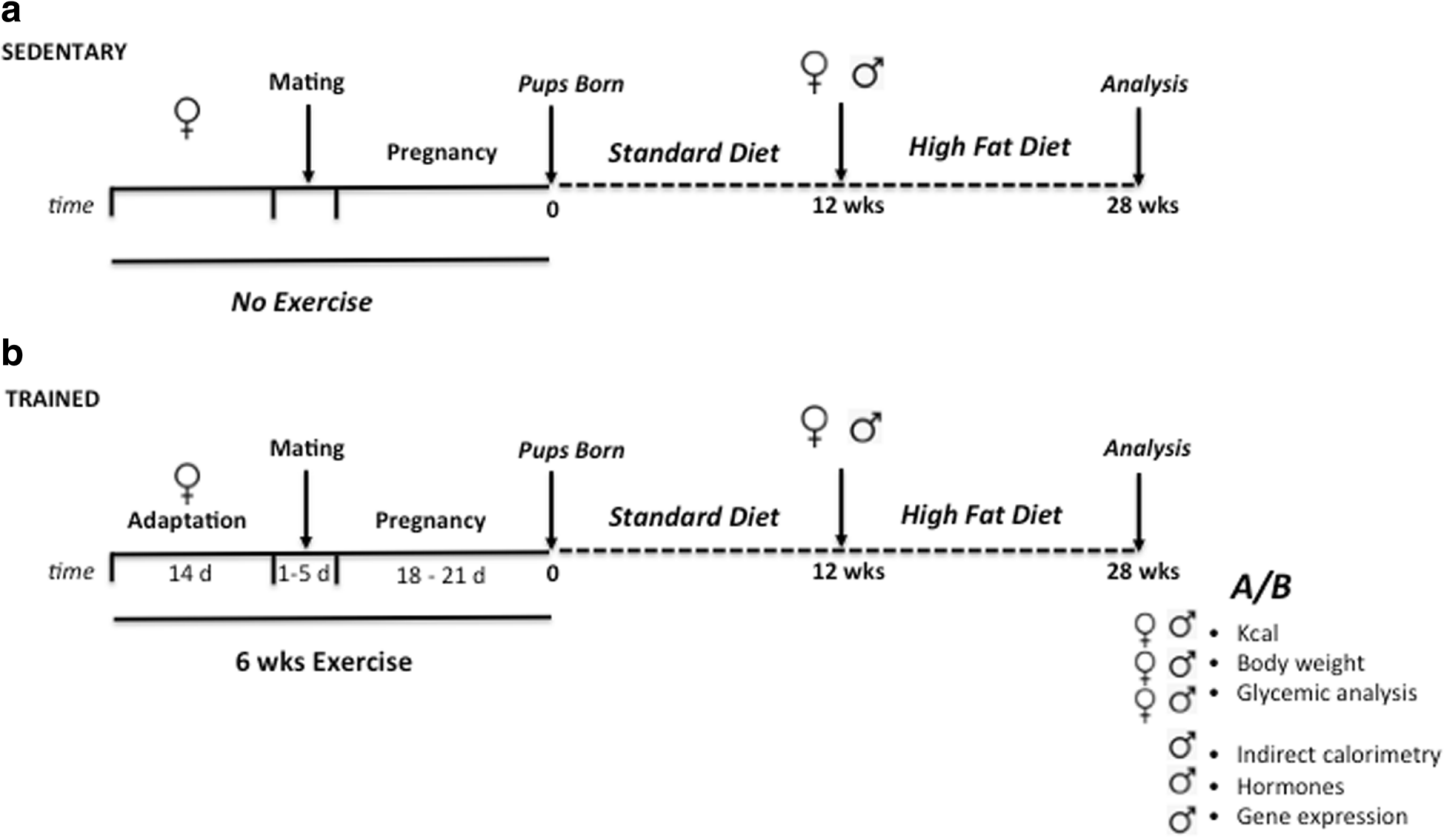
Experimental design. Female mice were either not subjected (**a**) or subjected (**b**) to an exercise adaptation period for 14 days followed by mating time (1-5 days). Group (**b**) was then exercising before and during pregnancy (1 h/day, 5 days/week) for 18-21 days (depending on the length of pregnancy). Male and female offspring of both groups was fed standard diet (SD) until 12 weeks of age. Afterwards, pups received HFD over a period of 16 weeks. At 28 weeks, animals were sacrificed and the tissue was used for a variety of analysis. (Notably, only male offspring was tested for indirect calorimetry, hormone levels and gene expression)

Female C57BL/6 mice (*n* = 18, age 8–12 weeks, 23–26 g) were obtained from the Animal Care Facility at the Federal University of São Paulo (UNIFESP), and single housed under standard conditions with access to water and food *ad libitum*. All procedures were previously reviewed and approved by the internal ethical committee of the institution. One group of 9 mice was subjected to swim training throughout a period of 6 weeks, and the other group of 9 females did not exercise (sedentary group, Fig. 1a). After a 2-week-adaptation period for the exercise group (1–14 days, Fig. 1b), animals were mated with males of the same strain. The presence of a vaginal plug was used to indicate pregnancy and was considered to be the first day of gestation (Figs. 1a–b). For the second part of the study, 3-month-old male and female offspring of either sedentary or trained mothers were submitted to HFD for 16 weeks. At the end of the experimental period, male and female offspring was sacrificed after an overnight fast and the tissue analyzed.

### Exercise protocol

Female animals were subjected to swim bouts in a swimming system that had been adapted for mice (containing 30 °C warm water) [14]. The 300-liter tank consisted of 10 lanes that were fitted with air pumps to maintain mice in constant motion. Swim training was performed during consecutive weekdays with 2 days off over the weekend. The adaptation period was comprised of 14 days with initial swim bouts of 20 min daily during the first week (day 1–5). The time gradually increased until mice were able to swim for 60 min daily at “moderate intensity” induced by a load (3 % of body weight attached to the tail) and according to our previous work [15]. Following 1–5 days of mating, the pregnant mice continued to exercise for 60 min per day (18 to 21 days corresponding to the last 3 weeks of the total exercise period, Fig. 1b); however, they were not loaded with extra weight during the last week of pregnancy.

### HFD in offspring

After weaning, male and female pups were separated and single housed receiving standard diet until 12 weeks of age (2.95 Kcal/g^−1^, 6 % calories from fat, 26.3 % protein, 67.7 % carbohydrates (Nuvilab mod. CR-1). For the 2^nd^ approach, 1 to 2 animals per mother were distributed to the groups fed standard diet (not shown) or HFD, sex-dependent (*n* = 6 pups of 4 mothers per group). To examine the amount of food intake, mice were single housed. Then, mice of both sedentary and trained mothers received HFD (‘*sedentary’ or ‘trained’ HFD*) consisting of 4.73 Kcal/g^−1^, 45 % calories from fat, 20 % protein, 35 % carbohydrates (Research Diets mod. D12451 New Brunswick, USA). Food and water intake was *ad libitum* and similar between groups. HFD was given for 16 weeks to induce obesity (Fig. 1a–b). During this period, animals were weighed weekly, and food intake was estimated by weighing leftover pellets.

### Glycemic analysis

A glucose tolerance test (GTT) and an insulin tolerance test (ITT) were performed twice, first at 12 weeks before the start of HFD (with unchanged results, data not shown), and second at the end of the experiment (at 28 weeks; Fig. 1a–b); in both occasions, animals had fasted for 12 h. To avoid stress, there was a 7-day interval between the GTT and ITT tests. Glycemia was assessed using a glucometer (Accu-Chek Advantage) that measures blood obtained from the tail vein. For the GTT and ITT, 1 g glucose per kg body weight (BW) and 1 IU of human recombinant insulin per kg BW, respectively, were injected intraperitoneally (i.p.). Glucose levels were determined at 0, 15, 30, 60, and 120 min after glucose injection and at 3, 15, and 30 min after insulin injection. The constant rate of glucose disappearance (K_ITT_) was calculated from the slope of the regression line obtained from log-transformed glucose values between 0 and 15 min after insulin administration according to current literature [16]. At 30 min, other hormone levels such as cortisol appear that would interfere with the result.

### Indirect calorimetry

Oxygen consumption, CO_2_ production, and respiratory quotient (RQ) were measured for all animals at 28 weeks and determined over 24 h with a LE405 gas analyzer (Panlab; Harvard Apparatus, Holliston, MA, USA). Animals were placed in chambers for 30 min per day, for 3 consecutive days before the measurements for acclimatization. Airflow was maintained using an Air & Supply Switching flow meter (Panlab; Harvard Apparatus, Holliston, MA, USA). The gas analyzer was calibrated using known concentrations of O_2_ and CO_2_ (Air Liquid, Sao Paulo, Brazil). Records for each mouse consisted of a 6 min interval every 30 min, and room air was used as reference. O_2_ and CO_2_ (VO_2_ and VCO_2_) flow was assessed using Metabolism version 2.2, expressed in mL.h-^1^.g based on the Withers equation and calculated using QR VCO_2_/VO_2_.

### Hormone measurements

A Milliplex Mouse Metabolic immunoassay was used at the end of the experiment at 28 weeks (in the morning of the following day) to analyze the levels of cytokines such as interleukin-6 (IL-6), resistin, C-peptide, insulin, leptin, and peptide YY (PYY). Mice were deeply anesthetized with ketamine/xylazine, and blood from the retro orbital venous plexus was collected. Blood was centrifuged at 1000 g for 10 min, and serum was taken for analysis. Tests were conducted as previously described by the manufacturer (Milliplex Mouse Metabolic Bead panel).

### Quantification of gene expression

White adipose tissue (WAT), brown adipose tissue (BAT), and skeletal muscle (SM) samples were taken at 28 weeks and frozen at -80 °C. Total RNA was isolated using TRIzol reagent (Invitrogen, Carlsbad, CA). First-strand cDNA was synthesized using the High Capacity cDNA Reverse Transcription kit (Applied Biosystems, Carlsbad, CA). Real-time PCR was performed using the TaqMan array kit in 96-well plates. The following genes related to fat/glucose metabolism or DNA methylation were evaluated: adiponectin (AdipoQ), which regulates metabolism and increases insulin sensitivity; NR3C1, a glucocorticoid receptor of the nuclear receptor subfamily 3, which is responsible for the expression of genes that are involved in metabolism, cellular development, and the immune response; Pparγ, or peroxisome proliferator-activated receptor, which acts in glycolytic metabolism, lipid uptake and adipogenesis; FAT/CD36, a fatty acid translocase that acts in lipid and glucose metabolism; Sirt1 (NAD-dependent deacetylase sirtuin-1) for which increased activity is associated with improved insulin sensitivity; Slc2a4, solute carrier family 2 (facilitated glucose transporter) member 4, also known as GLUT4; Pck1 (Phosphoenolpyruvate carboxykinase 1), which is a main control point for gluconeogenesis; Dnmt1, DNA (cytosine-5)-methyltransferase 1 and Dnmt3L, (cytosine-5)-methyltransferase 3-like, which are both involved in DNA methylation.

mRNA levels of leptin in SM were measured using the following primers: (5′-AGGAGAACCAAGCAACGACA-3′ forward and 5′-CGTTTTTCCATCTTCTTCTTTG-3′ reverse), and UCP-1 expression in BAT was measured using the following primers: 5′-AGCCGGCTTATGAACTGGA3′ forward and 5′-CGTGTAGCGGGGTTTGAT-3′ reverse. Expression levels of the genes of interest were normalized to β-Actin.

### Statistical analysis

To evaluate significant differences, the data were analyzed by student’s *t*-test. All of the values are expressed as the mean ± SDM. *P* values <0.05 were considered to be statistically significant.

## Results

### Smaller offspring body weight following swim exercise

**Fig. 2:**
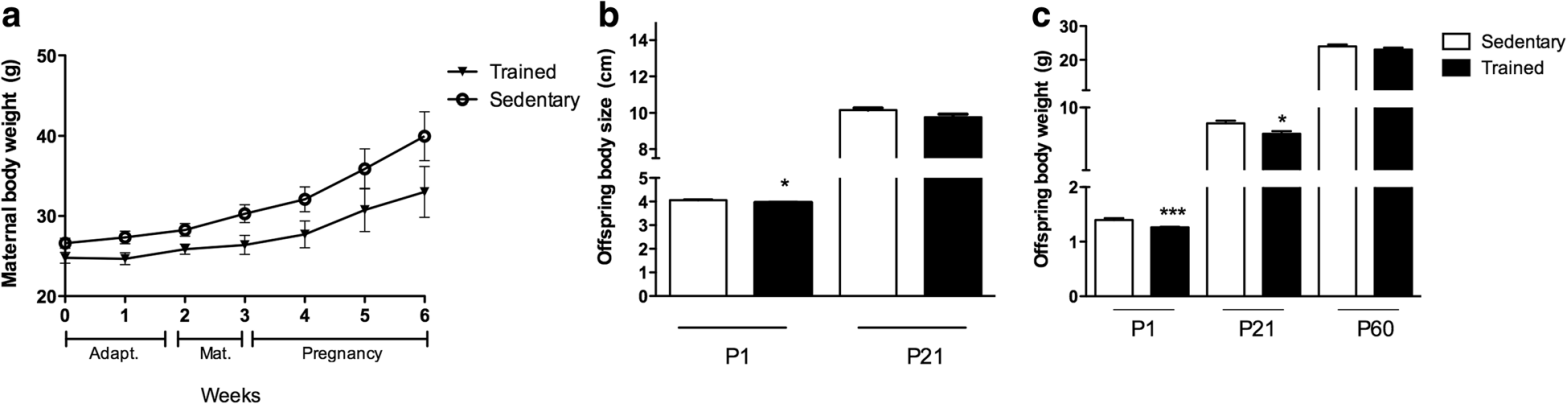
Maternal body weight, offspring size and weight. **a** Maternal body weight before as well as during mating and pregnancy did not change upon exercise (*n* = 9 per group) (**a**). **b**–**c** Both, offspring size (**b**) and weight (**c**) from exercising mothers (black bar) was significantly decreased at postnatal day (P) 1 (male and female pups pooled). At weaning (P21), only the weight of the offspring was still decreased (male pups). At P60, no difference in the weight of male offspring was observed (**c**). Data are presented as mean ± SDM. Open bar: offspring of sedentary mothers. *p < 0.05, ***p < 0.001

We first determined maternal body weight and the number of offspring for both exercised and sedentary mothers (Fig. 2). Maternal body weight similarly increased through the gestational period in both groups (Fig. 2a) with no effect of training on the number of offspring per mother (in average 6 to 7 mice). Yet, animals of exercised mothers were born smaller (P1 sedentary 4.06 ± 0.03 cm vs. trained 3.95 ± 0.02 cm, male and female pups, *p* = 0.018; Fig. 2b) compared with those from sedentary mothers, and they weighed significantly less (P1 *p* = 0.0002; Fig. 2c). At weaning (P21), animals of both groups did not present significant differences in body size (only male pups were analyzed; sedentary 10.15 ± 0.64 cm vs. trained 9.76 ± 0.90 cm, *p* = 0.06; Fig. 2b); however, pups from swimming mothers still weighed significantly less (male mice, sedentary 7.98 ± 1.35 g vs. trained, 6.64 ± 1.3 g, *p* = 0.009; Fig. 2c). At P60, male pups born from trained mothers revealed the same weight (Fig. 2c).

### No increase in body weight following HFD in offspring of exercising mothers

**Fig. 3:**
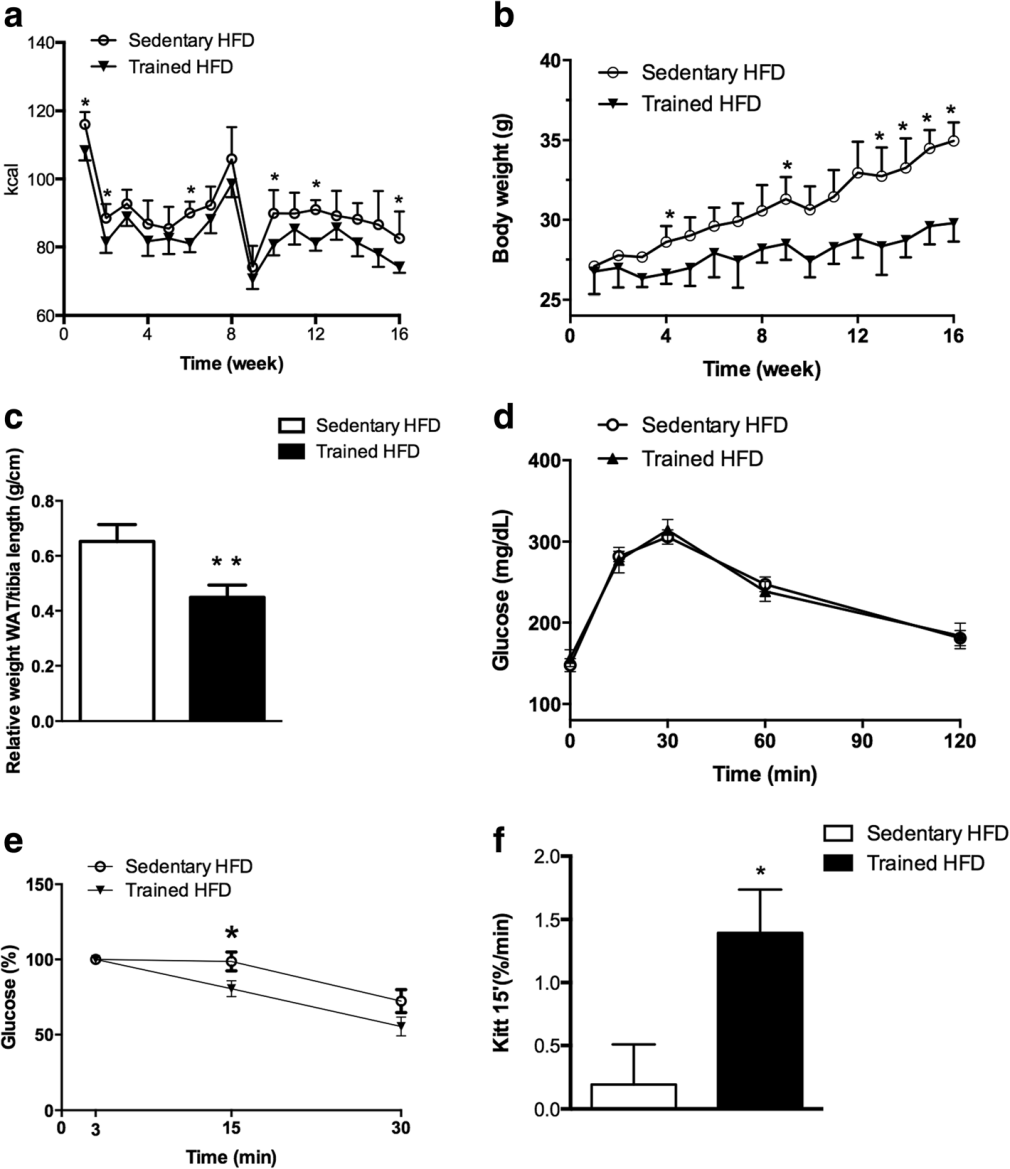
Food intake and glucose metabolism of male offspring submitted to HFD. **a** Calorie intake (Kcal) over 16 weeks was reduced in trained HFD group. **b** Male offspring of exercising mothers revealed less body weight throughout the experiment. **c** Relative white adipose tissue (WAT, inguinal) normalized to tibia length (cm) was decreased in trained HFD group (black bar). **d** No differences between groups were observed in Glucose tolerance test (GTT). However, the decline in glucose uptake after insulin stimulation (ITT) was lower in trained HFD group (**e**). **f** Constant rate of glucose disappearance (Kitt) calculated from ITT displayed increased levels in the offspring of exercising mothers. Data are presented as mean ± SDM *p < 0.05; **p < 0.01 (*n* = 6 per group)

We next investigated energy intake and body weight of adult male and female offspring during 16 weeks of HFD. Both, energy intake and weight gain was less in male and female mice of trained mothers during the last weeks of HFD compared with their sedentary counterparts (female, *p* < 0.05; Additional file 1: Figure S1A-B), with male offspring (Fig. 3) displaying constantly less weight throughout the experiment (Fig. 3b). Since the reduction in caloric intake and protection against weight gain was more evident in male offspring, the additional experiments were performed using only male mice. In accordance to body weight and caloric intake, male animals from exercising mothers displayed less WAT fat mass (normalized to tibia length, *p* = 0.0001; Fig. 3c) that corresponded to reduced fat mass/g BW (sedentary 0.036 ± 0.007 g vs. trained 0.028 ± 0.008 g, *p* = 0.029) in comparison to mice of sedentary mothers.

We next determined changes in glucose metabolism following HFD in offspring of exercising and sedentary mothers at 28 weeks. An increase in blood glucose in response to GTT was observed in male offspring fed HFD of both sedentary and trained mothers (Fig. 3d). Levels are increased in comparison to standard diet (data not shown). However, a time-dependent decline in plasma-glucose following ITT was significantly lower in male mice of sedentary mothers fed HFD compared with the trained group (*p* = 0.03; Fig. 3e). Female mice submitted to HFD did not present differences in GTT (Additional file 1: Figure S1C) and ITT (Additional file 1: Figure S1D). Note, these results contributed to our decision to follow up with only male mice. K_itt_ analysis in male offspring revealed the same reduction in insulin sensitivity for the sedentary HFD group (*p* = 0.04; Fig. 3f). Thus, male animals of swimming mothers were unaffected by HFD and insulin sensitive.

### Increased energy expenditure but less spontaneous activity in HFD-fed offspring of trained mothers

**Fig. 4:**
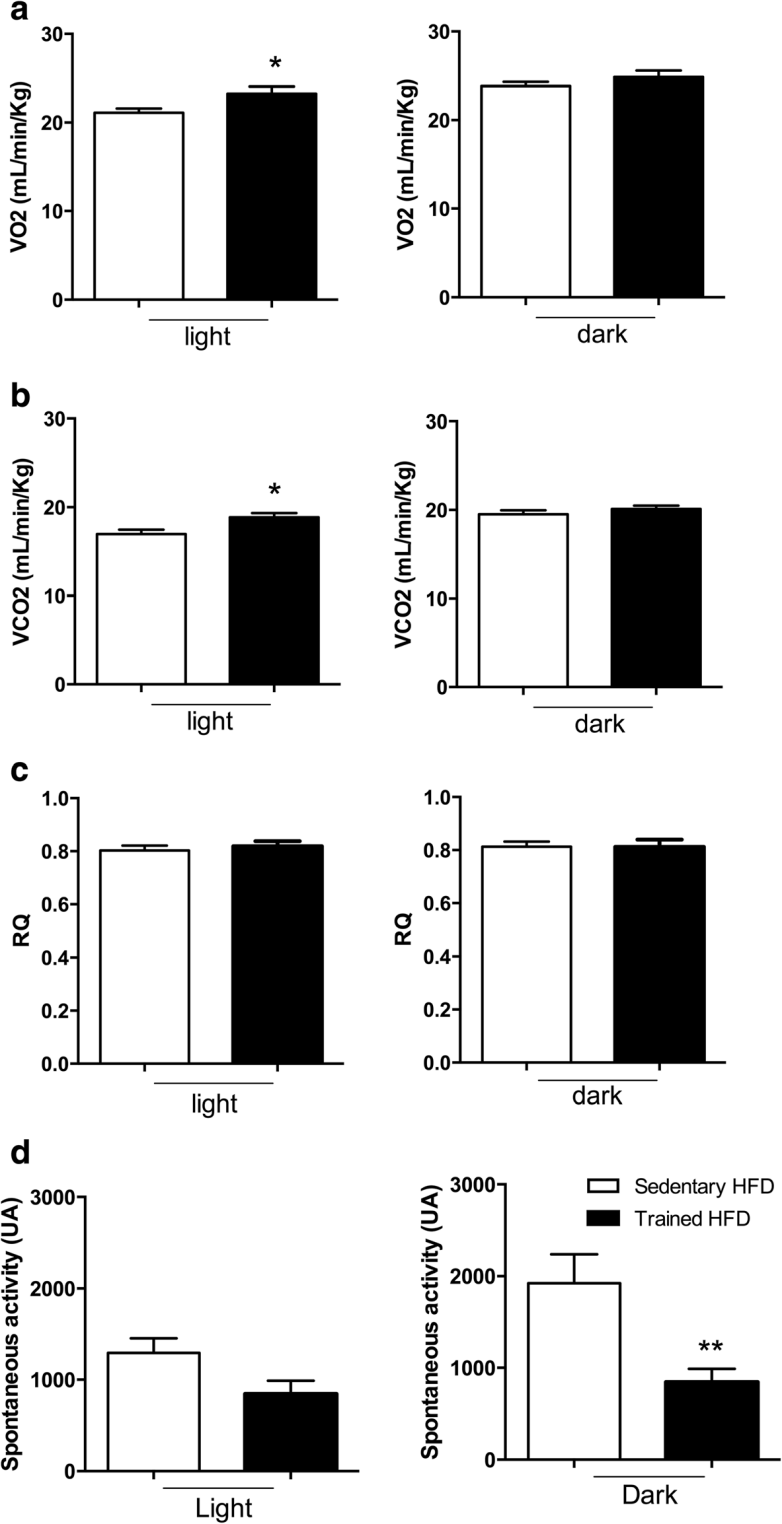
O_2_/CO_2_ consumption/production in male offspring following HFD. **a**–**b** VO_2_ consumption (**a**) and VCO_2_ production (**b**) is increased in male offspring of trained mothers (black bar). **c** No changes in the Respiratory quotient (RQ). **d** Less spontaneous activity in the offspring of trained mothers was observed (black bar) at night. Data are presented as mean ± SDM. *p < 0.05, **p < 0.01 (*n* = 6 per group)

We next asked whether the smaller offspring weight and size from exercised mothers influenced the offspring’s metabolism, and measured O_2_ consumption/CO_2_ production. Offspring of trained mothers fed HFD for 4 months displayed increased VO_2_ and VCO_2_ during the day-light phase, whereas no difference was observed at night (Figs. 4a–b) at 28 weeks. Despite the increase in VO_2_ and VCO_2_, the proportion of carbohydrate and lipid oxidation (RQ) was similar between mice of sedentary and trained mothers during the light and dark periods (Fig. 4c). Surprisingly, when spontaneous activity was determined, male mice of trained mothers were less active compared with mice of sedentary mothers at night, whereas no difference was observed during the light period (measured by calorimetry; Fig. 4d).

### Increased PYY levels and decreased inflammation

A positive correlation between dietary fat and body weight gain is well documented in the rodent model. Increased consumption of fat-enriched food can alter the levels of peptides and hormones (e.g., insulin and leptin), which in turn modulate food intake and induce secretion of inflammatory cytokines such as TNF- α and IL-6 from adipose tissue and endocrine organs [17]. Thus, to determine differences in adiposity between groups in this study, we investigated serum levels of several hormones (C-peptide, insulin, PYY, resistin, and leptin) and IL-6 using Milliplex analysis (Table 1). No differences were observed in serum hormone levels of C-peptide, insulin, resistin and leptin. However, HFD in the offspring of trained mothers increased PYY (sedentary 138 ± 28 vs. trained 208 ± 52; *p* = 0.040; Table 1). Statistical analysis revealed a significant decrease in IL-6 in trained HFD offspring compared with sedentary HFD (sedentary 52 ± 12 vs. trained 35 ± 5; *p* = 0.019; Table 1).

**Table 1:** Cytokine and hormone levels in male offspring after 16 weeks of HFD (pg/ml)

	Sedentary HFD	Trained HFD	*p*-values
C-Peptide	1315 ± 461	1516 ± 999	ns
Insulin	1026 ± 229	1513 ± 285	ns
PYY	138 ± 28	208 ± 52*	0.040
Resistin	15041 ± 1549	16045 ± 2175	ns
Leptin	17110 ± 1832	16881 ± 8882	ns
IL-6	52 ± 12	35 ± 5*	0.019

*p < 0.05, between HFD groups; students *t*-test was used for individual comparison; *HFD* high-fat diet

### Maternal swim exercise positively affects AdipoQ and Dnmt3l expression in adult offspring

**Fig. 5:**
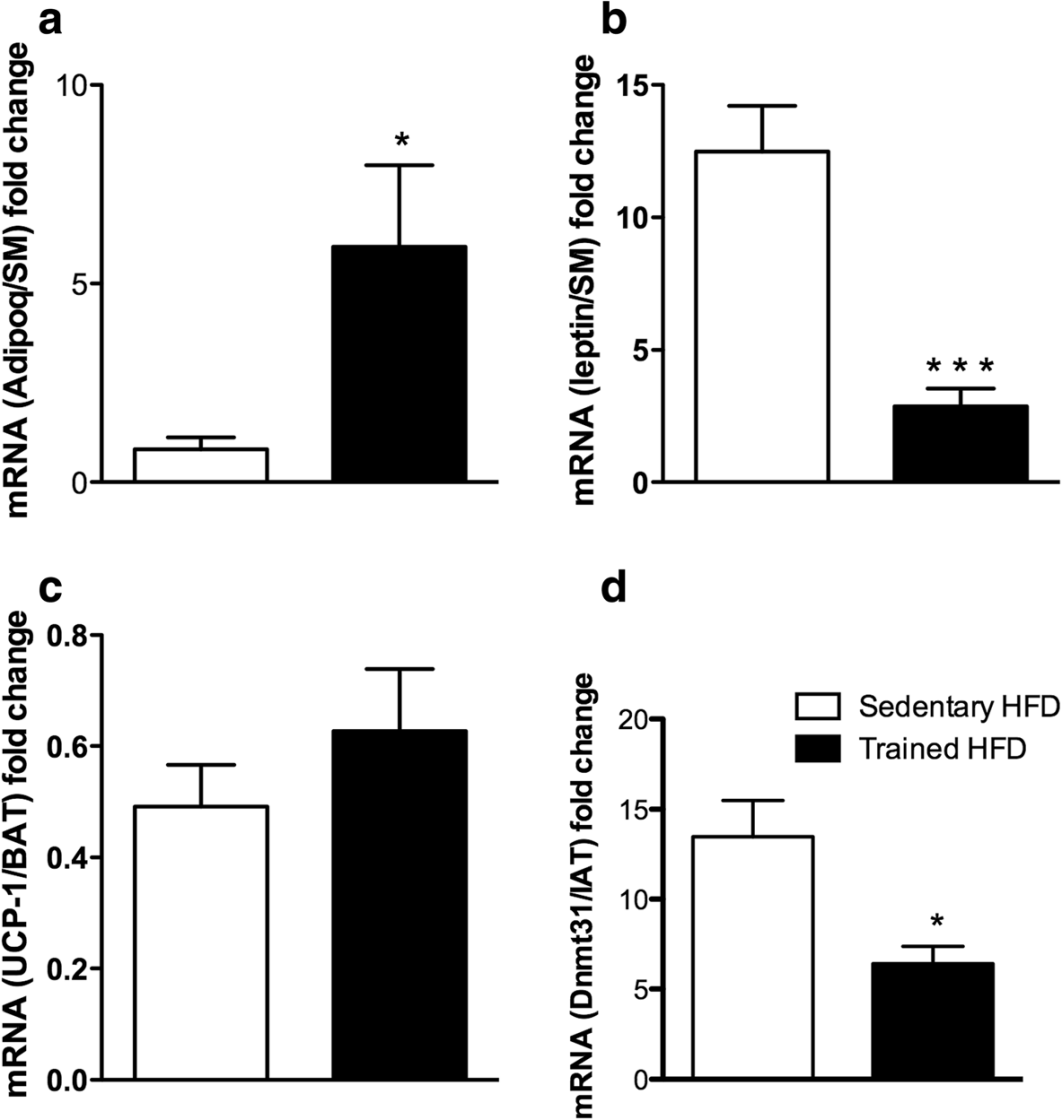
Analysis of adiponectin and leptin expression in skeletal muscle (SM), Dnmt3L expression in white adipose tissue, and UCP-1 expression in brown adipose tissue after HFD. **a** AdipoQ levels were increased in male offspring of trained mothers (black bar). **b** Leptin levels were significantly decreased in trained HFD group (black bar). **c** UCP-1 expression was unchanged, while (**d**) Dnmt3L was decreased in the trained HFD group (black bar). Data are presented as means ± SDM. *p < 0.05 (*N* = 6)

Given the importance of WAT, BAT, and SM to energy expenditure and nutrient disposal, we investigated whether maternal exercise before and during pregnancy affected the expression of genes related to metabolism and DNA methylation in these tissues. The expression of several genes was evaluated in WAT, BAT, and SM of sedentary and trained groups (Additional file 2: Table S1). Only a few genes revealed significant alterations in expression. In SM, AdipoQ expression levels were significantly increased in the trained HFD group (*p* = 0.022; Fig. 5a and Additional file 2: Table S1). Notably, we also measured serum AdipoQ levels, and there were no significant differences between the groups (data not shown). Significantly lower leptin mRNA levels were observed in the trained HFD group (*p* = 0.0008; Fig. 5b). Thus, increased leptin mRNA levels that occur following HFD in sedentary control pups (a 6-fold increase was observed in comparison to SD, data not shown) were not seen in the trained HFD group. When mitochondrial-uncoupling protein (UCP) 1 was measured after HFD, no differences in UCP-1 expression were observed in BAT (sedentary 0.49 ± 0.07 vs. trained 0.62 ± 0.11; *p* = 0.342; Fig. 5c). In WAT, validation of Dnmt31 revealed decreased expression levels in the trained HFD group compared with the sedentary HFD group (sedentary 13.0 ± 2.0 vs. trained 6.4 ± 0.96; *p* = 0.012; Fig. 5d). Generally, Dnmt3L levels were increased after HFD in offspring of sedentary mothers compared with the SD (data not shown.)

## Discussion

Our data demonstrate that continuous swim exercise in mice before and during pregnancy leads to beneficial effects in the adult male offspring submitted to HFD. First, we demonstrated that maternal swim exercise positively affected both offspring size and weight, which is an outcome that varies in the literature with respect to different exercise models [18–20]. Second, when exposed to HFD for 16 weeks, the adult offspring of trained mothers had lowered increase in body and relative WAT weight with an overall increase in basal metabolism. We propose that this may be due to the lower energy intake (Kcal) consumed by mice of trained mothers at several data points in addition to a consequent resistance to body weight gain resulting from maternal training. Furthermore, our data demonstrate that maternal swim exercise also prevents high glucose levels (determined by ITT and K_iTT_) in the adult that had been fed HFD. We also observed changes in circadian rhythm (increased oxygen consumption/CO_2_ production during light phase); yet, mitochondrial activity was unaffected.

We present new results describing how swimming can affect and protect adult offspring from HFD-induced obesity later in life. We did not observe behavioral differences in mothers after withdrawal of exercise that would impact feeding or maternal care. Initially, the experiments for HFD were performed in female and male mice; however, our findings demonstrated that female offspring was less influenced by exercise practice during pregnancy. Significant sexual differences in response to exercise have been shown earlier [21] which may result of acute exercise bouts effects that differ between men and women; e.g. in response to exercise, women have relative greater reliance of fat spend as energy during [22, 23] and after exercise [21]. Although a few data exist regarding the mechanisms that promote the different adaptations between sexes in response to exercise, our data and the literature permit us to speculate that changes in the uterus environment occur following exercise.

Recent studies in rats and mice mainly focused on consequences of exercise such as running, and/or diabetes of mothers during pregnancy [24] on the offspring. They demonstrated improved glucose homeostasis in offspring of maternal running [12, 13] that may lead to a decrease in the development of obesity. Our data for the first time show that maternal swimming may reduce the risk to develop high fat diet-related diseases in the offspring. Other consequences are altered gene expression or reduced hypermethylation [25]; epigenetic factors are discussed in humans [26, 27]. However, the overall metabolic effects of maternal exercise on the offspring are not well understood; nevertheless, the beneficial outcome to the next generation is evident. Fetal life is highly influenced by a detrimental environment of nutrition and oxygen to the growing fetus [28]. The consequences are adverse fetal growth pattern as well as development of metabolic syndrome in adult life [28]. On the other hand, short- and long-term exercise during pregnancy seems to affect genes able to induce metabolic changes that could be beneficial for the offspring [29–31]. Although we did not evaluate DNA-methylation per se, our results confirm the hypothesis that exercise induces alterations in gene expression. In our study, the effect of HFD in adulthood was determined, and improved peripheral insulin sensitivity (ITT) was observed; however, serum insulin levels did not change in offspring of exercised mothers. The effect of maternal exercise on improved glucose tolerance may depend on the offspring’s age [13].

Another factor in insulin sensitivity is the large SM tissue, which is important for total body energy expenditure/homeostasis [32]. SM releases both adiponectin and leptin [33], and altered levels are indicators for obesity. Increased AdipoQ expression in SM in our study implies that there are positive effects of swimming during pregnancy and emphasizes the importance of adiponectin in regulation of metabolism and insulin sensitivity. Maternal swim exercise also prevents a normally exponential increase in leptin with fat mass in SM (as was observed for sedentary HFD in our study). Leptin is another hormone that plays a role in energy homeostasis, which is increased by insulin [34–36]. It has been hypothesized that leptin resistance in obesity could promote SM atrophy while the recovery of its function could reverse the loss of muscle mass [37]. In humans, long-term exercise can chronically reduce serum leptin levels [38]. Our data reveal that leptin serum levels were unchanged in offspring of exercising mothers. Conversely, decreased SM leptin levels were observed, which was accompanied by less insulin following HFD in adult offspring of swimming mothers. We conclude that maternal exercise leads to long-term changes in adult offspring with benefits for basal metabolism that protects from HFD.

While investigating other hormones that were related to metabolism and obesity, increased PYY serum levels after HFD were observed in adult offspring of maternal swimmers, which is a potential indicator for inhibitory appetite effects [39]. In accordance, these animals had lower caloric intake suggesting that increased PYY expression led to positive changes in the metabolism. Serum analysis demonstrated less inflammation by decreased IL-6 concentrations in the trained HFD group. Levels of this cytokine are increased in obesity, which may contribute to the pathogenesis of insulin resistance [40, 41]. In our study, less inflammation goes hand in hand with improved insulin sensitivity. Furthermore, our data reveal lower levels of Dnmt3L expression that directly interacts with the catalytic domains of Dnmt3a and Dnmt3b, which in turn act as regulatory factor of DNA methylation [42]. Transgenic mice with Dnmt3a overexpression display increased inflammation after HFD that is accompanied by increased expression of the tumor necrosis factor-α (TNF-α) and the monocyte protein-1 (MCP-1) [43]; increased Dnmt3a expression may also contribute to inflammatory-associated obesity. However, maternal exercise protected the adult offspring from diet-induced obesity after HFD. DNA methylation has also been linked to obesity as an epigenetic factor (e.g., in SM). Yet, our results suggest adaptations in the SM as observed by alterations in AdipoQ and leptin levels.

Changes in insulin sensitivity after HFD may also affect circadian rhythms in rodents and humans. Studies reveal dysfunction in the circadian clock after HFD, which lead to obesity as well as insulin resistance during the daily phase of inactivity [44, 45]. However, in our study, no changes in VO_2_/VCO_2_ and RQ have been observed in the sedentary group fed HFD. Yet, circadian rhythm was altered in the offspring of swimming mothers, which reveals unchanged spontaneous activity from day to night (the most active phase in rodents) and increased oxygen consumption O_2_/CO_2_ production during the day. Thus, the overall energy expended was increased during an entire 24 h-period, arguing for better obesity protection. Furthermore, offspring from swimming mothers presented lower spontaneous activity at night. One could speculate that training promotes a depressive-like behavior, and some studies argue that animals rather struggle to survive instead of performing a mere physical effort in water [46]. This is particularly relevant when considered as acute exposition to a water environment as has been shown in the (Porsolt) forced swim test (FST) [47, 48]. In our study, we did not test for depression-like behavior since we have been focusing on metabolism. Female mice were chronically exposed to water (the struggle to survive is reduced by repeated exposition to water); thus, they would not show the same degree of stress in comparison to animals exposed to FST. In contrary, studies on swimming mice for 4 weeks presented improvements in a model of Parkinson disease when assessed depressive-like behavior, locomotor behavior and long-term memory; accompanied with increased levels of interleukin 1-beta and of reactive oxygen species [49]. According to these studies the effects of exercise were due to its antioxidant and anti-inflammatory properties. Our findings fit to this in that continuous swim exercise is able to increase immunity, to counterbalance the effects of experimental arthritis and of inflammation of adipose tissue promoted by HFD as shown earlier [15, 50, 51].

Regarding alterations in circadian rhythm, adiponectin is a likely candidate that can peripherally influence the circadian clock as it improves oxidative metabolism in SM. Treadmill running of rodents in daylight led to adaptations in SM, and animals were less active at night [52, 53]. Transgenic mice expressing human adiponectin in liver demonstrated higher activity during daylight, thus altering the circadian rhythm of locomotor activity [44]. Decreased adiponectin plasma levels as observed in obese patients strongly correlate with various components of metabolic syndrome [54–56]. A potential role of adiponectin in energy metabolism is the stimulation of mitochondrial biogenesis [57, 58]. However, no changes in UCP-1 expression were observed in our study.

It is important to note that several studies reporting negative effects of a lower birth weight did not involve exercise; in addition, weight reduction reported after caloric restriction and exercise was much higher than observed here. Despite a lower birth weight, our data provide additional evidence that the risk to develop obesity remains reduced in the adult when HFD is provided. Obesity, type 2 diabetes (T2D), metabolic syndrome, and cardiovascular diseases are physical states influenced by exercise. Several studies report an impact of exercise on cardiometabolic function in T2D, irisin levels, and mitigation of some effects of HFD [59]. Thus, the relevance and potential implications of obesity in adult offspring are enormous considering the intake of higher levels of fat per diet in an occidental society. Considering this, we expect our data a relevant contribution to this field. Nevertheless, due to the complexity of our study, given gender differences for part of the experiments, single housing, quantity, and time of the work and facts determined, animal numbers are small and additional experiments need to be done.

## Conclusions

Overall, our study indicates that maternal swim exercise is beneficial and affects basal metabolism in adult male offspring that was submitted to HFD over a long period of time. As main targets, SM and WAT have altered gene expression and hormone levels. Glucose tolerance and energy expenditure were improved, thereby protecting the offspring from HFD-induced obesity. Our data support swim exercise during pregnancy since it has positive effects on weight and energy homeostasis.

## Additional files

Additional file 1:
**Food intake and glucose metabolism of female offspring submitted to HFD.** (A-B) Calorie intake (Kcal; A) as well as body weight (B) were significantly reduced at the end of 16 weeks measured in trained HFD group. (C-D) No differences between groups were observed in the Glucose tolerance test (GTT, C) and when glucose uptake was determined after insulin stimulation (ITT, D). Data are presented as mean ± SDM **p* < 0.05; ***p* < 0.01 (*n* = 6 per group). (TIF 5527 kb) Additional file 2:
**Expression of several genes related to fat/glucose metabolism and DNA methylation in male offspring after 16 weeks of HFD.** (DOCX 24.4 kb)

## Abbreviations

BAT: Brown adipose tissue
GTT: Glucose tolerance test
HFD: High fat diet
ITT: Insulin tolerance test
Kitt: Constant rate of glucose disappearance
RQ: Respiratory quotient
SM: Skeletal muscle
WAT: White adipose tissue
