# Supplementary figures and images for: Exercise during pregnancy protects adult mouse offspring from diet-induced obesity
Source: Nutr Metab (Lond). 2015 Dec 18;12:56. doi: 10.1186/s12986-015-0052-z (PMC4683957; doi:10.1186/s12986-015-0052-z)

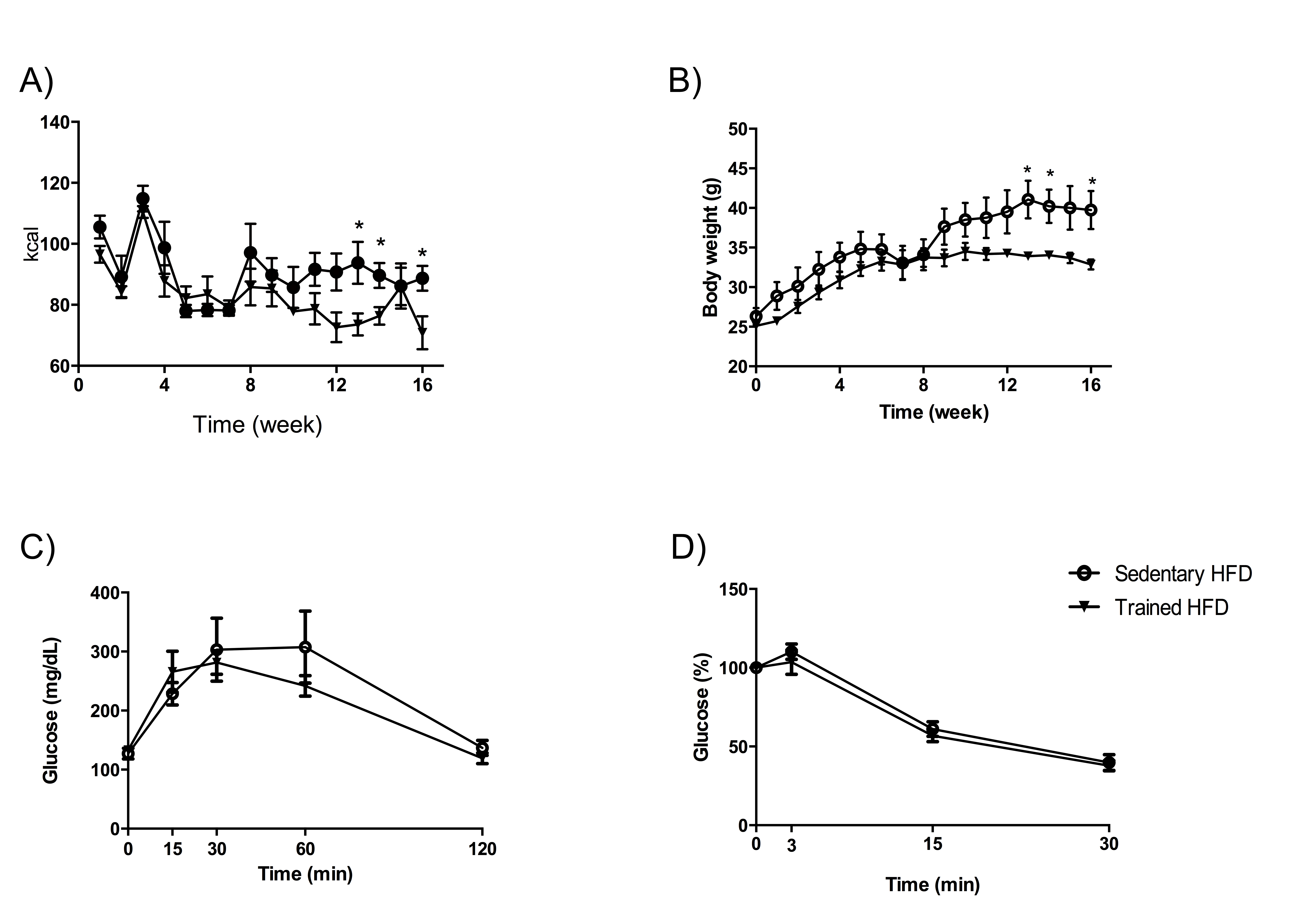

Supplement: Additional file 1: — Food intake and glucose metabolism of female offspring submitted to HFD. (A-B) Calorie intake (Kcal; A) as well as body weight (B) were significantly reduced at the end of 16 weeks measured in trained HFD group. (C-D) No differences between groups were observed in the Glucose tolerance test (GTT, C) and when glucose uptake was determined after insulin stimulation (ITT, D). Data are presented as mean ± SDM *p < 0.05; **p < 0.01 (n = 6 per group). (TIF 5527 kb) [file 12986_2015_52_MOESM1_ESM.tif]
