# Supplementary material for: Exercise during pregnancy protects adult mouse offspring from diet-induced obesity
Source: Nutr Metab (Lond). 2015 Dec 18;12:56. doi: 10.1186/s12986-015-0052-z (PMC4683957; doi:10.1186/s12986-015-0052-z)
Supplement: Additional file 2: — Expression of several genes related to fat/glucose metabolism and DNA methylation in male offspring after 16 weeks of HFD. (DOCX 24.4 kb) [file 12986_2015_52_MOESM2_ESM.docx]

**Additional file 2: Table S1.**

**Expression of several genes related to fat/glucose metabolism and DNA methylation in male offspring after 16 weeks of HFD.**

| Regulation of metabolism | | | | | Insulin and glucose signaling | | | | DNA methylation | |
| --- | --- | --- | --- | --- | --- | --- | --- | --- | --- | --- |
|  | gene | Adipoq | Nr3c1 | Pparγ | Cd36 | Sirt1 | Slc2a4 | Pck1 | Dnmt1 | Dnmt31 |
| WAT |  |  |  |  |  |  |  |  |  |  |
| Sedentary HFD | (N=6) | 0.59 ± 0.09 | 0.61 ± 0.06 | 0.66 ± 0.12 | 1.54 ± 0.13 | 0.32 ± 0.09 | 0.88 ± 0.19 | 0.25 ± 0.08 | 0.59 ± 0.13 | 13 ± 2.00 |
| Trained HFD | (N=6) | 0.44 ± 0.08 | 0.52 ± 0.03 | 0.44 ± 0.03 | 1.20 ± 0.37 | 0.23 ± 0.04 | 1.30 ± 0.24 | 0.27 ± 0.07 | 0.49 ± 0.10 | **6.4 ± 0.96*** |
| P value |  | NS | NS | NS | NS | NS | NS | NS | NS | 0.01 |
| BAT |  |  |  |  |  |  |  |  |  |  |
| Sedentary HFD | (N=6) | 0.67 ±0.04 | 0.95 ± 0.17 | 1.12 ± 0.11 | 0.80 ± 0.14 | 0.88 ± 0.26 | 0.88 ± 0.08 | 0.79 ± 0.12 | 1.38 ± 0.12 | 8.68 ± 1.47 |
| Trained HFD | (N=6) | 0.72 ±0.13 | 0.93 ± 0.12 | 1.45 ± 0.30 | 0.93 ± 0.14 | 1.03 ± 0.13 | 0.82 ± 0.09 | 0.92 ± 0.13 | 1.25 ± 0.12 | 6.64 ± 0.07 |
| P value |  | NS | NS | NS | NS | NS | NS | NS | NS | NS |
| SM |  |  |  |  |  |  |  |  |  |  |
| Sedentary HFD | (N=6) | 0.82 ± 0.30 | 1.45 ± 0.25 | 3.77 ± 1.04 | 1.75 ± 0.28 | 1.22 ± 0.07 | 1.71 ± 0.92 | 1.10 ± 0.24 | 1.43 ± 0.20 | 0.16 ± 0.04 |
| Trained HFD | (N=6) | **5.93 ± 2.06*** | 1.42 ± 0.22 | 4.82 ± 1.71 | 1.71 ± 0.24 | 1.07 ± 0.12 | 4.22 ± 1.79 | 2.68 ± 1.09 | 1.54 ± 0.25 | 0.31 ± 0.13 |
| P value |  | p<0.05 | NS | NS | NS | NS | NS | NS | NS | NS |

WAT- white adipose tissue; BAT- brown adipose tissue; SM- skeletal muscle; HFD- high-fat diet. Data are presented as means ± SEM. *p<0.05 between HFD groups.
